# Feasibility properties of the EQ-5D-3L and 5L in the general population: evidence from the GP Patient Survey on the impact of age

**DOI:** 10.1186/s13561-022-00374-y

**Published:** 2022-05-20

**Authors:** Ole Marten, Wolfgang Greiner

**Affiliations:** School of Public Health, Department of Health Economics and Health Care Management, Bielefeld University, Universitaetsstrasse 25, Bielefeld, Germany

**Keywords:** EQ-5D-3L, EQ-5D-5L, Health-related quality of life, Feasibility, Older population, GPPS

## Abstract

**Background:**

There is evidence to suggest that the proportion of missing values is slightly higher in the older population resulting in lower completion rates of the EQ-5D. However, existing studies rarely provide a within-sample comparison of feasibility properties across age groups to quantify this difference. Hence, this study examines feasibility properties of the EQ-5D-3L and 5L in the general population and explores the impact of age on the completion of EQ-5D instruments.

**Methods:**

We pool five waves from the English GP Patient Survey, where respondents self-report their health in either EQ-5D-3L or 5L. Descriptive analysis was undertaken to analyse the distribution and proportion of missing values and completion rates stratified by age and EQ-5D version; logistic regression models were specified to quantify the impact of age, gender and potential long-term conditions on the completion of each of the EQ-5D instruments.

**Results:**

The total sample comprises ~ 4.36 million observations, of which 2.88 million respondents report their health in 5L and 1.47 million in 3L, respectively. Respondents over 64 years have slightly more missing values in each dimension than younger respondents. The highest share was observed for the oldest age group in the dimension anxiety/depression (3L 9.1% vs. 5L 7.6%), but was otherwise below 5%. Consequently, completion rates (observed and predicted) decreased with older age and at a higher rate after the age of 64; this was more pronounced for the 3L.

**Conclusion:**

Evidence from our study suggests that both the EQ-5D-3L and 5L have good feasibility properties. In comparison to younger populations there appears to be a higher proportion of respondents with incomplete responses beyond the age of 64 years. Overall, the 5L version compares more favourably in terms of missing values, completion rates as well as with regard to the expected probability of an incomplete descriptive system.

**Supplementary Information:**

The online version contains supplementary material available at 10.1186/s13561-022-00374-y.

## Background

Over the past decades the consideration of the patient perspective has gained enormous importance for health care and policy decision making [1–3]. With regard to this, the measurement of self-reported health-related quality of life (HRQoL) is a major component on this pathway. The EQ-5D is a well-established and widely used instrument develop by the EuroQol Group specifically intended for that purpose [4]. Over the years the EQ-5D has developed a significant role in economic decision making, since prominent health technology assessment bodies in the United Kingdom, the Netherlands, Spain or France recommend or clearly specify that HRQoL should be measured using the EQ-5D [5, 6]. Since the release of the EQ-5D-5L – with five response levels - [7], extensive research has been conducted to compare the measurement properties of the EQ-5D-3L and EQ-5D-5L (hereafter 3L and 5L, respectively). A recent literature review confirms that both the 3L and the 5L are applicable to a wide range of populations, while confirming improved informativity, less ceiling effects and better distributional properties for the 5L [8]. Further studies examined the measures’ feasibility, which was commonly operationalised in terms of missing values and completion rates at the individual level. The former is either defined as unit nonresponse or as item nonresponse, where information is unavailable for the respondent as a whole or just on individual items [9]. Whereas the latter construct is defined as the share of computable EQ-5D index values, which requires complete information on all five items of the descriptive system [10]. Studies by Janssen et al. [11] and Agborsangaya et al. [12] reported very good feasibility of both 3L and 5L with missing values of less than 2%, whereas Buchholz et al. [8] conclude on a proportion of missing values of less than 5% from reviewing 15 studies.

However, there is evidence to suggest that the proportion of missing values is slightly higher in the older population resulting in lower completion rates of the instrument [10]. Even though studies conducted with older respondents [13–17] report missing values well within the margin reported by Buchholz et al. [8], there are other studies reporting proportions of up to 10% [18–21]. On the contrary, the samples in Janssen et al. [11], Agborsangaya et al. [12] and other studies [22–24] were younger than 65 years and reported considerable fewer missing values. Hence, we suspect that the share of missing responses and incomplete descriptive systems may be driven by an age-dependent effect, as this was also described for the SF-36 [25–27]. Terwee et al. [28] argue that missing values may be indicative of problems with item interpretability, which is confirmed by findings from Hulme et al. [19] and van Leeuwen et al. [29] who report this kind of response issues on the 3L for older people.

Hence, this study aims to assess the magnitude of missing values and incomplete responses for both the 3L and 5L using five waves of the large-scale General Practitioner Patient Survey (GPPS) based on age-stratified comparisons of these feasibility parameters in the English general population, which allows a within-sample assessment of differences between the older and the younger general public.

## Methods

### Data

For the analysis we utilise individual-level self-reported EQ-5D data from the GPPS [30]. This is a large-scale cross-sectional survey undertaken on behalf of the National Health Service (NHS) England. Since 2007 the survey is sent yearly to more than 2 million adults asking them about their experience with their general practitioner and other NHS services. The questionnaire is primarily posted to participants; however, the survey can also be completed online or by telephone. Moreover, it is available in a variety of languages. The overall GPPS samples are obtained by drawing proportionately stratified samples from each practice using registration data held by the NHS Digital database. Individuals are eligible for inclusion in the survey, if they are 18 years or above, hold a valid NHS registration number and were continuously registered with a general practitioner (GP) for at least 6 months [31]. Even though respondents are recruited via GP registries, we would like to argue that the underlying sample is in effect recruited from the general population, since registration with a GP does not necessarily imply that respondents are currently under treatment for a condition or an illness.

### Variables

The primary aim of the GPPS survey is to assess patients’ experiences with their GP and other local NHS services; questions include aspects such as access to services, appointments, waiting times and how people manage their health. In addition to that, respondents answer the EQ-5D, which has been used in the survey between 2011 and 2017 [31]. The EQ-5D is a standardised generic measure of HRQoL developed by the EuroQol Group. The EQ-5D descriptive system entails five dimensions: mobility, self-care, usual activities, pain or discomfort and anxiety or depression. The initially developed EQ-5D-3L has three response levels, allowing respondents to describe their health status based on three options: no problems (level 1); some or moderate problems (level 2); or extreme problems/unable to (level 3) [32]. The EQ-5D-5L is a re-developed version covering the same five dimensions, but expanding the available response options to five levels, again, ranging from no problems (level 1), over slight, moderate and severe problems to extreme problems/unable to (level 5) [7]. The response from each dimension-level can be concatenated to form a health profile, which can be linked to a value set – a scoring algorithm with preference-based weights for each dimension-level – to generate a single summary index score [33]. The second component of the EQ-5D - the visual analogue scale (EQ VAS) – is not included in the GPPS survey [34].

In this study, we pool data from several years. We use data from 2012, as this is the last year the 3L was used, also capturing the effect of changing the instrument to the 5L between the first and the second wave of 2012. Further, we examine data from 2016 and 2017, since this was the most recent data when we applied for the data set. The survey mode was equivalent across all 3 years in the sense that the vast majority of respondents answered the questionnaire paper-based and only 4–6% of the respondents answered using the online survey, while telephone responses were negligible [30, 35–38]. Since the publicly available analysis tool does not allow in depth examination of all EQ-5D data, we submitted an application for individual-level data to NHS England. Further, we were granted access to reported background information, which is based on gender, age groups and existence of any of the following long-term conditions: Alzheimer’s disease/ dementia, angina/heart problem, arthritis/joint problem, asthma/chest problem, blindness, cancer, deafness, diabetes, epilepsy, high blood pressure, kidney or liver disease, long-term back problem, long-term mental health problem or long-term neurological problem [31]. The information on the administration mode was not included in the individual-level data set and, hence, could not be controlled for.

### Analysis

We examine feasibility of the EQ-5D in older persons in comparison to the general population by investigating distributional properties of EQ-5D data as well as the prevalence and distribution of missing values, which ultimately prevent the calculation of an EQ-5D index value. We do so by conducting descriptive analysis based on the proportion of respondents per level in both 3L and 5L for the whole sample as well as stratified by age groups. We expect to observe a lower proportion of level 1 responses (i.e. at the ceiling) on the 5L in general and more pronounced in respondents aged 65 and above.

As suggested by Janssen et al. [11], we examine feasibility for both 3L and 5L in terms of missing values separately for each dimension and stratified for age groups. We further report completion rates based on the same criteria. We analyse the proportion of missing values by age groups using chi-square tests to examine potential associations with age. Given the large-scale of this exercise, we report standardised effect sizes based on Cramer’s V to quantify the magnitude of observed differences [39]. We further explored the impact of age, gender and having a long-term health condition on the probability of returning an incomplete EQ-5D using logistic regression analysis. We used “incomplete response” as a binary dependent variable where 1 indicates that at least one EQ-5D item was not answered and, thus, we were unable to calculate an index value. We used ‘female’ and ‘condition’ as binary independent variables, where 1 represents being female or having a long-term condition, respectively. Further, age group was added as a categorical variable into the model with 18–24 years as the reference category. We used STATA’s *margins* post-estimation command to calculate predicted probabilities of returning an incomplete EQ-5D for each age group holding the other variables at their sample means. We apply the conventional significance level of 5%. All analysis was conducted using STATA 16 [40].

## Results

### Sample description

After pooling five different waves of the GPPS the total sample comprised 4,358,700 observations. Of those, 1,476,395 contributed to the 3L sample, whereas 2,882,305 respondents were represented in the 5L sample. As Table 1 suggests, the sample characteristics were similar across the 3L and 5L sample including slightly more women. About one third of the sample was 65 years and above (3L: 33.8%; 5L: 36.7%) and about 60% reported at least one long-standing health condition. The most prevalent long-term condition was high blood pressure (23%) followed by arthritis or joint problems (16%). Mental health problems including Alzheimer’s disease/dementia and neurological problems were reported by 15.9 and 15.5% for the 3L and 5L sample, respectively.

**Table 1:** Sample characteristics of five waves of GP Patient survey data

Characteristics	EQ-5D-3L sample		EQ-5D-5L sample		Total	
	N	%	N	%	N	%
Gender						
Male	636,076	43.1	1,255,846	43.6	1,891,922	43.4
Female	840,324	56.9	1,626,459	56.4	2,466,783	56.6
Age group						
18–24 years	65,729	4.5	116,338	4.0	182,067	4.2
25–34 years	148,966	10.1	267,430	9.3	416,396	9.6
35–44 years	203,616	13.8	363,407	12.6	567,023	13.0
45–54 years	259,698	17.6	501,875	17.4	761,573	17.5
55–64 years	300,329	20.3	577,672	20.0	878,001	20.1
65–74 years	273,922	18.6	595,753	20.7	869,675	20.0
75–84 years	169,686	11.5	347,876	12.1	517,562	11.9
85 or over	54,454	3.7	111,954	3.9	166,408	3.8
Long-standing health condition						
Yes	895,175	60.6	1,771,617	61.5	2,666,792	61.2
No	535,210	36.3	1,022,939	35.5	1,558,149	35.8
Don’t know/ can’t say	27,722	1.9	58,320	2.0	86,042	2.0
Missing	18,293	1.2	29,429	1.0	47,722	1.1
Long-term condition						
Alzheimer/ dementia	9929	0.7	21,101	0.7	31,030	0.7
Angina/ heart problems	96,707	6.6	179,424	6.2	276,131	6.3
Arthritis/ joint problems	240,480	16.3	466,970	16.2	707,450	16.2
Asthma/ chest problems	147,325	10.0	297,099	10.3	444,424	10.2
Blindness/ visual problems	18,500	1.3	32,530	1.1	51,030	1.2
Cancer in the last 5 yrs	54,362	3.7	118,398	4.1	172,760	4.0
Deaf/ hearing problems	70,311	4.8	139,745	4.9	210,056	4.8
Diabetes	123,405	8.4	266,795	9.3	390,200	9.0
Epilepsy	15,531	1.1	28,152	1.0	43,683	1.0
High blood pressure	337,554	22.9	668,507	23.2	1,006,061	23.1
Kidney or liver problems	25,649	1.7	56,508	2.0	82,157	1.9
Long-term back problems	157,361	10.7	306,127	10.6	463,488	10.6
Long-term mental health problems	51,121	3.5	119,783	4.2	170,904	3.9
Long-term neurological problems	26,744	1.8	60,278	2.1	87,022	2.0
Long-term other health problems	172,282	11.7	366,061	12.7	538,343	12.4

### Comparison of response distribution

Tables 2 and 3 provide an overview of the response distribution for each dimension stratified by age groups for both the 3L and 5L, respectively. Unsurprisingly, problems were always least prevalent in the youngest age groups with a monotonically increasing trend with increasing age. Problems were more frequently reported when using the 5L and limitations were spread wider across the severity range. Generally, self-care appears to be the least affected dimension with a considerable ceiling effect. Even in the highest age group only 40% report any problems with self-care, whereas 82% report problems in mobility and pain or discomfort in that age group. Interestingly, problems with pain or discomfort and anxiety or depression were the most frequent in younger age groups (around 30% vs. self-care 5% vs. mobility 9%). While limitations in pain or discomfort increase considerably with age, the proportion of any reported problems in anxiety or depression remains fairly stable; this pattern is constant across both EQ-5D versions. Overall, floor effects, where respondents respond with the worst answer category, are not observable in this general population sample. Severe and extreme problems are least prevalent in the dimensions self-care and anxiety or depression. However, while severe and extreme problems with self-care increase with age the opposite seems to be the case for anxiety or depression. Again, this pattern is consistent across both the 3L and 5L, with the exception being level 3 in mobility in the 3L (‘confined to bed’), which was the least frequent overall.

**Table 2:** Distribution of EQ-5D-5L responses by dimension and age group

Parameter		Total	Age group Proportion in %							
**Dimension**	**Level**	N	18–24	25–34	35–44	45–54	55–64	65–74	75–84	85+
**Mobility**	No problems	1,958,750	91.2	90.2	86.2	78.5	68.6	58.8	39.6	18.0
	Slight problems	403,522	4.6	5.0	7.0	10.5	14.7	18.9	23.9	23.0
	Moderate problems	268,905	1.6	1.8	2.9	5.3	8.5	12.2	20.3	29.2
	Severe problems	170,235	0.6	0.8	1.6	3.4	5.7	7.3	12.1	22.0
	Unable to	22,630	0.4	0.4	0.4	0.5	0.6	0.7	1.3	4.1
**Self-care**	No problems	2,484,621	94.9	94.7	92.9	89.6	86.2	84.3	76.6	60.2
	Slight problems	149,380	1.7	1.8	2.5	3.7	5.0	6.1	9.5	15.0
	Moderate problems	118,583	1.0	1.0	1.6	3.0	4.4	5.0	7.2	12.5
	Severe problems	41,437	0.4	0.4	0.7	1.2	1.7	1.6	2.0	4.7
	Unable to	20,059	0.4	0.4	0.4	0.4	0.5	0.5	1.2	4.3
**Usual activities**	No problems	1,943,526	85.3	85.4	81.8	75.0	67.7	61.4	45.3	24.6
	Slight problems	446,180	7.8	7.9	9.5	12.6	15.9	19.3	24.3	24.3
	Moderate problems	259,886	3.3	3.0	4.0	6.1	8.6	10.9	17.2	25.0
	Severe problems	114,562	1.2	1.3	1.9	3.1	4.4	4.5	6.4	11.5
	Unable to	55,120	0.6	0.6	0.8	1.1	1.5	1.7	3.9	10.9
**Pain/Discomfort**	No	1,272,899	72.4	68.6	60.3	49.3	40.3	33.6	24.6	18.0
	Slight	874,407	18.2	20.7	24.9	29.7	33.2	33.6	34.6	31.2
	Moderate	460,993	5.6	6.4	8.7	12.4	16.1	20.0	26.7	33.8
	Severe	171,788	1.5	2.0	3.1	4.9	6.8	7.3	9.5	11.3
	Extreme	39,224	0.4	0.6	1.0	1.6	1.8	1.4	15	2.0
**Anxiety/Depression**	No	1,879,193	67.0	68.6	67.5	64.5	64.5	67.3	62.0	53.6
	Slight	556,159	17.3	17.7	18.4	19.4	19.4	19.0	20.7	24.1
	Moderate	252,616	9.0	8.0	8.0	9.2	9.4	7.8	8.9	12.4
	Severe	61,933	3.1	2.4	2.4	2.9	2.6	1.3	1.2	1.6
	Extreme	30,483	1.7	1.3	1.4	1.6	1.2	0.5	0.5	0.7

**Table 3:** Distribution of EQ-5D-3L responses by dimension and age group

Parameter		Total	Age group Proportion in %							
**Dimension**	**Level**	N	18–24	25–34	35–44	45–54	55–64	65–74	75–84	85+
**Mobility**	No problems	1,074,533	92.7	92.0	88.2	82.0	73.2	63.6	45.5	23.1
	Some problems	362,930	4.9	5.7	9.3	15.6	24.5	33.6	51.0	72.5
	Confined to bed	4545	0.3	0.3	0.2	0.2	0.2	0.2	0.5	1.7
**Self-care**	No problems	1,307,043	95.6	95.5	93.8	91.4	88.8	86.2	79.9	64.7
	Some problems	118,593	2.0	2.2	3.7	6.0	8.4	10.2	14.3	25.0
	Unable to	11,759	0.4	0.4	0.4	0.4	0.5	0.7	1.6	5.5
**Usual activities**	No problems	1,061,308	88.1	87.9	83.6	77.8	71.6	66.3	51.3	30.4
	Some problems	332,279	8.9	9.2	12.8	18.0	23.7	27.9	38.5	49.6
	Unable to	47,921	0.9	1.0	1.5	2.2	2.8	3.4	6.5	15.6
**Pain/Discomfort**	No	780,144	73.1	76.3	68.8	58.5	49.2	40.8	29.9	22.6
	Moderate	571,456	17.5	19.8	25.6	33.7	41.6	49.0	57.5	63.1
	Extreme	84,005	1.3	1.7	3.2	5.4	7.0	7.2	8.4	9.6
**Anxiety/Depression**	Not	1,057,588	78.6	77.7	74.4	70.7	70.8	71.9	66.8	59.3
	Moderately	314,019	16.1	17.2	19.4	22.3	22.5	20.9	23.4	29.5
	Extremely	38,506	2.7	2.6	3.2	3.7	3.0	1.5	1.4	2.1

### Feasibility of the EQ-5D-3L and 5L

Table 4 summarises the share of missing values and completion rates by age groups based on the 3L and 5L. Overall, the proportion of missing values in any of the EQ-5D dimensions was very low but increasing with age. Chi-square tests suggest that the proportion of missing values in any dimension are not independent of the respondents’ age (*p* < 0.001). Given the large sample size, this test result is not surprising and mitigated by the negligible association (Cramér’s V). Nonetheless, it appears as if there is a steeper increase in the last two age categories. The highest proportions were found in anxiety or depression for respondents 65 years and above, where the proportion of missing responses peaks at 7.6% (5L) and 9.1% (3L) for the oldest respondents. Apart from this, the proportion of missing responses is less than 5% across all dimensions, and generally lower for the 5 L in comparison to the 3L. Missing value patterns stratified for age groups can be found in the Appendix (see Appendix Tables A1 and A2). Among those respondents with missing values, patterns with just one missing item account for ~ 60–70% depending on age and EQ-5D version. Moreover, patterns with two to four missing responses only accumulate between 22 up to 30% of respondents with missings. Interestingly, the proportion of complete non-response to both 3L and 5L is highest among young adults (18–24 years - 5 L: 19.8%; 3L: 18.4%) and drastically decreases with higher age (85 years and over - 5 L: 2.8%; 3L: 2.6%).

**Table 4:** Proportion of missing values and overall EQ-5D completion rate stratified by age and EQ-5D version

Parameter	Missing	Age group Proportion in %								Cramér’s V
EQ-5D-5L	N (%)	**18–24**	**25–34**	**35–44**	**45–54**	**55–64**	**65–74**	**75–84**	**85+**	
Mobility	58,263 (2.02)	1.61	1.68	1.89	1.88	1.89	2.04	2.71	2.72	0.0229
Self-care	68,225 (2.37)	1.66	1.72	1.98	2.07	2.22	2.57	3.52	3.37	0.0363
Usual activities	63,034 (2.19)	1.85	1.88	2.06	2.00	1.94	2.11	2.98	3.76	0.0310
Pain/Discomfort	62,994 (2.19)	1.82	1.82	2.05	2.03	1.93	2.08	3.05	3.78	0.0328
Anxiety/Depression	101,921 (3.54)	1.98	2.01	2.37	2.46	2.89	4.12	6.78	7.56	0.0897
Completion rate	93.11%	96.01	95.89	95.18	94.68	94.06	92.24	87.78	85.92	0.1103
EQ-5D-3L										
Mobility	34,391 (2.33)	2.10	2.05	2.24	2.18	2.07	2.53	3.00	2.77	0.0205
Self-care	39,003 (2.64)	1.98	1.97	2.21	2.20	2.27	2.89	4.22	4.86	0.0496
Usual activities	34,890 (2.36)	2.06	2.03	2.15	2.01	1.87	2.43	3.68	4.42	0.0441
Pain/Discomfort	40,795 (2.76)	2.19	2.18	2.48	2.42	2.29	2.98	4.26	4.61	0.0439
Anxiety/Depression	66,287 (4.49)	2.66	2.54	2.99	3.28	3.79	5.69	8.36	9.14	0.0963
Completion rate	91.57%	94.88	94.91	93.98	93.39	92.70	89.83	85.20	83.14	0.1199

Further, we report completion rates based on age groups, i.e. proportion of respondents with all five items completed (see also Table 4). Across all age groups the 5L completion rate was found to be higher in comparison to the 3L and for both completion was negatively associated with age, however, this effect was very weak. While on average only 4% of the 5L utilities cannot be calculated in the youngest age group, this figure increases to more than 14% in those 85 years and above. Correspondingly, these figures range from 5% (18–24 years) to 16.8% (85+ years) for the 3L.

**Fig. 1:**
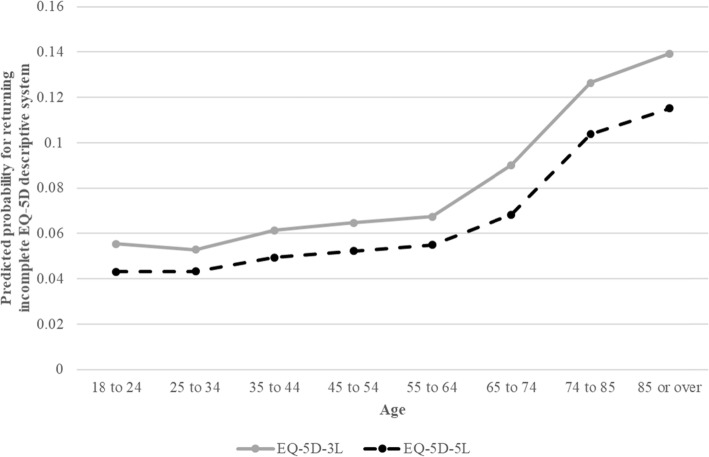
Predicted probabilities for returning incomplete EQ-5D-3L or 5L based on age groups using logistic regression

Figure 1 presents predicted probabilities for returning an incomplete 3L or 5L for each age group controlling for gender and the presence of any reported long-term condition. Firstly, the probability of an incomplete EQ-5D response was lowest in the age group 25–34 years for the 3L (5.3%) and for the 5L (4.3%) in those 18–24 years, respectively. Up until the age of 64, the probability only marginally increased by 1.2 percentage points for both the 3L and 5L. However, beyond the age of 64 years the probability of an incomplete EQ-5D response accelerated quickly peaking at 13.9% for the 3L and 11.5% for the 5L in those being 85 years and above. Secondly, the probability of an incomplete response was found to be lower at any given age for the 5L in comparison to the 3L. The difference was between 1.0 (25–34 years) and 2.4 (85 or over) percentage points (see Fig. 1) with the spread being wider after the age of 64 years.

## Discussion

The aim of this study was to assess feasibility of the 3L and 5L for older respondents in direct comparison to younger adults in the general population. The 5L showed better feasibility than the 3L across all age groups. The superiority of the 5L was more noticeable in older age groups, which was indicated by fewer missing values, higher completion rates and an overall lower likelihood of an incomplete response to the descriptive system.

With respect to the descriptive system we observe an improved response distribution when measured with the 5L as compared to the 3L version. As one might expect, the proportion of respondents at the ceiling is lower on the 5L and further decreasing with increasing age. This finding is consistent with other studies comparing the 3L and 5L version [8, 11]. Similarly, the response distribution in individual dimensions in the older population was also similar to that found in earlier studies, where self-care is the least informative dimension, whereas pain or discomfort is the most informative. Again, the response distribution in anxiety or depression does not seem to be moderated by age in both 3L and 5L [41–43].

The cross tabulation of incomplete responses per dimension with age categories clearly shows an increasing trend in higher age groups (Table 4). Generally, our findings are in line with proportions of missing values reported earlier [11, 12]. Especially in younger age groups we rarely observe more than 2% missing values per dimension, whereas the proportions are only marginally higher in older age and fall well within the overall margin reported in Buchholz et al. [8]. As an exception to this rule, we observed a higher proportion of missing values in the dimension of anxiety or depression, which go as high as 7.6% (5L) and 9.1% (3L) in those being 85 years and over. Holland and colleagues [44] report that this dimension caused some embarrassment in older respondents. This may partially explain the higher prevalence of missing values in that particular dimension. Moreover, we found that in each dimension and for any given age group the proportion of missing responses was lower for the 5L – even though the difference was less than half a percent. This findings are also in line with earlier studies [8, 17, 45].

While the share of missing response was relatively low at the dimension-level, the completion rate, i.e. the ability to generate the utility value from the respondent’s reported health state, was just above 90% on the total sample for both 3L and 5L. A potential explanation for this observation may be that missing values resulted from relatively many individuals with just one missing item rather than from respondents with multiple missing responses, which was suggested by the analysis of missing value patterns (see Appendix Tables A1 and A2). Moreover, completion of the 3L and 5L decreased by approximately 10 percentage points from the youngest to the oldest age group and more rapidly after the age of 64 years. For the total sample, we find that our observed completion rates for the 3L and 5L are about 5 percentage points lower than those reported in other studies in the general population [12, 46–49]. Considering the older population, 3L completion rates were 5–10 percentage points lower than those reported in a study from Switzerland, which also reports age group-specific completion [50]. However, the differences in the data collection process may largely account for this variance, since Luthy et al. [50] used computer-assisted personal interviews to collect data instead of self-reports as was the case in this sample. Overall, literature on the feasibility properties of the 3L and 5L in the general public is scarce, where completion rates are predominantly reported for the overall sample. We are unaware of other studies providing age-specific completion rates, which limits further comparison with our findings.

We further provide predicted probabilities for an incomplete response based on a logistic regression model controlling for age groups, gender and presence of a long-term condition. The predicted probabilities largely follow the pattern from the uncontrolled cross-tabulations (Table 4) confirming the hypothesis of an age-dependent impact on the EQ-5D’s completion with a more pronounced effect beyond the age of 64 years. Importantly, the 5L performs better than the 3L in the sense that the predicted probability of returning an incomplete descriptive system is consistently lower for the 5L. Evidence from the literature suggests that the length of the response scale has an effect on the data quality [51]. The question-answer process may be distorted, if the intended response does not match the available response options, which may cause the respondent to refuse to answer [29, 52]. Hence, it may be assumed that the lack of sensitivity in the 3L is in part responsible for the higher prevalence of missing values in the descriptive system, since respondents may lack the ability to report an appropriate level of problems on the three-level scale. Therefore, the improved sensitivity of the 5L [53, 54] may lead to improved feasibility as well, which is supported by our findings of better feasibility of the 5L in terms of reduced missing values, higher completion rates and lower probability of reporting an incomplete descriptive system. This notion is further underpinned by findings from Janssen and colleagues [55], where respondents argued that the 5L was easier to use and better reflects their response in comparison to the 3L.

Depending on the purpose of future studies, the slightly higher propensity for missing responses among aged respondents may have different implications. If the aim is to collect HRQoL data in a limited sample and to calculate quality-adjusted life years, researchers could consider assisted or interviewer-based approaches to mitigate the risk of bias due to incomplete response from older respondents [13, 56, 57]. At the same time, applying interviewer-based approaches may come at the cost of introducing other types of biases such as interviewer effects, socially desirable answers or a reduced willingness to disclose sensitive information, which may trade-off the gains of increased completeness [58]. However, on an aggregate level, such as in a population health survey like the GPPS, the extent of missing values can be rated as good or negligible [8, 11]. Nevertheless, our results suggest that missing values vary systematically by age groups and were more prevalent in older adults. This ultimately implies a bias in estimated utility values against older respondents, which needs to be addressed adequately in statistical analyses.

A strength of our study is the huge sample size, which we gained by pooling data from several years of a consistent population health survey. In addition to that, we are able to compare responses to the descriptive system of both versions of the EQ-5D and across all age groups, however, it was not possible to compare the 3L and 5L on a like-for-like comparison, since respondents did not complete both measures. Due to the origin of the data, we had no information on how independently respondents answered the EQ-5D, i.e. whether respondents may have received help filling in the questionnaire and, hence, the level of feasibility problems for a self-report survey may be underestimated. A major limitation of our study is the missing EQ VAS component, which was not included in the survey and, hence, we were unable to investigate its feasibility properties. An in-depth analysis of the EQ VAS’ feasibility properties in the general population seems desirable, since it is known to present problems to older adults [10]. Similarly, a qualitative study may facilitate a better understanding of the differences in feasibility properties between the 3L and 5L, which would also be welcomed for the EQ VAS. Future research should further explore the impact of different administration modes, i.e. paper-based vs. online completion, as we were not able to control for this factor even though the sample size would have been sufficient. Additionally, the GPPS data may allow an in-depth exploration od the impact of different long-term conditions on completion of both EQ-5D versions.

## Conclusion

Evidence from our study suggests that both the 3L and 5L have good feasibility properties. The proportion of missing values is acceptable and low across all age groups. However, in comparison to younger populations there appears to be a higher proportion of respondents with incomplete responses, thus resulting in lower completion rates. Predicted probabilities for an incomplete response significantly increased beyond the age of 64 years for both versions of the EQ-5D, indicating a higher likelihood of missing values. Generally, we conclude that either version of the EQ-5D is applicable and feasible in the older population. However, the 5L version compares more favourably in terms of missing values, completion rates as well as with regard to the expected probability of an incomplete descriptive system.

## Supplementary Information

**Additional file 1.**

## Data Availability

The data that support the findings of this study are available from NHS England but restrictions apply to the availability of these data, which were used under license for the current study, and so are not publicly available. Data are however available from the authors upon reasonable request and with permission of NHS England.

## Abbreviations

3L: EQ-5D-3L
5L: EQ-5D-5L
EQ VAS: EQ-5D visual analogue scale
GP: General practitioner
GPPS: General Practitioner Patient Survey
HRQoL: Health-related quality of life
NHS: National Health Service
