# Supplementary material for: Feasibility properties of the EQ-5D-3L and 5L in the general population: evidence from the GP Patient Survey on the impact of age
Source: Health Econ Rev. 2022 May 20;12:28. doi: 10.1186/s13561-022-00374-y (PMC9121571; doi:10.1186/s13561-022-00374-y)
Supplement: Supplementary file 1 — Additional file 1. [file 13561_2022_374_MOESM1_ESM.docx]

**Feasibility properties of the EQ-5D-3L and 5L: Evidence from the GP Patient Survey**

Appendix

Table A1: Distribution of EQ-5D-5L profile missing value patterns by age groups.

| Pattern | Missings | EQ-5D-5L | | | | | | | |
| --- | --- | --- | --- | --- | --- | --- | --- | --- | --- |
|  |  | 18-24 years | 25-34 years | 35-44 years | 45-54 years | 55-64 years | 65-74 years | 75-84 years | 85 or over |
| + + + + - | 1 | **18.60%** | **17.27%** | **18.57%** | **20.33%** | **24.35%** | **29.15%** | **32.87%** | **31.03%** |
| + + + - + | 1 | **11.07%** | **11.91%** | **12.17%** | **12.59%** | **11.03%** | **10.20%** | **9.38%** | **10.26%** |
| + + - + + | 1 | **13.61%** | **12.59%** | **11.05%** | **10.63%** | **9.82%** | **9.18%** | **8.74%** | **11.45%** |
| + - + + + | 1 | **6.22%** | **6.51%** | **6.95%** | **8.74%** | **10.07%** | **10.18%** | **9.74%** | **9.03%** |
| - + + + + | 1 | **7.67%** | **8.49%** | **8.26%** | **8.68%** | **8.84%** | **8.64%** | **8.45%** | **8.10%** |
| + - + + - | 2 | **0.58%** | **0.93%** | **1.17%** | **1.63%** | **2.58%** | **3.97%** | **4.23%** | **3.45%** |
| + + + - - | 2 | **2.26%** | **2.22%** | **2.51%** | **2.39%** | **2.24%** | **2.61%** | **3.59%** | **4.99%** |
| - - + + + | 2 | **2.17%** | **2.31%** | **2.59%** | **2.78%** | **2.52%** | **2.16%** | **1.62%** | **1.09%** |
| + + - + - | 2 | **0.90%** | **1.06%** | **1.01%** | **1.08%** | **1.09%** | **1.46%** | **2.00%** | **2.73%** |
| + - - + + | 2 | **0.75%** | **1.04%** | **1.10%** | **1,16%** | **1.35%** | **1.26%** | **1.28%** | **1.17%** |
| - + + + - | 2 | **0.50%** | **0.53%** | **0.63%** | **0.73%** | **0.98%** | **1.29%** | **1.33%** | **1.43%** |
| + + - - + | 2 | **1.12%** | **0.82%** | **0.96%** | **1.02%** | **0.79%** | **0.71%** | **0.91%** | **1.11%** |
| - + + - + | 2 | **0.86%** | **0.71%** | **0.91%** | **0.88%** | **0.86%** | **0.91%** | **0.87%** | **0.81%** |
| - + - + + | 2 | **0.37%** | **0.57%** | **0.54%** | **0.61%** | **0.65%** | **0.59%** | **0.70%** | **0.85%** |
| + - + - + | 2 | **0.43%** | **0.46%** | **0.58%** | **0.67%** | **0.66%** | **0.53%** | **0.65%** | **0.67%** |
| + - - + - | 3 | **0.39%** | **0.63%** | **0.85%** | **0.97%** | **1.19%** | **1.37%** | **1.38%** | **1.07%** |
| - - - + + | 3 | **0.99%** | **1.10%** | **1.66%** | **1.62%** | **1.42%** | **1.00%** | **0.73%** | **0.48%** |
| - - + + - | 3 | **0.45%** | **0.70%** | **0.76%** | **0.88%** | **1.00%** | **0.99%** | **0.79%** | **0.43%** |
| + + - - - | 3 | **0.60%** | **0.86%** | **0.86%** | **0.76%** | **0.68%** | **0.55%** | **0.81%** | **1.43%** |
| + - + - - | 3 | **0.30%** | **0.31%** | **0.29%** | **0.39%** | **0.41%** | **0.54%** | **0.65%** | **0.86%** |
| - - + - + | 3 | **1.10%** | **0.61%** | **0.60%** | **0.65%** | **0.66%** | **0.37%** | **0.24%** | **0.24%** |
| - + + - - | 3 | **0.39%** | **0.34%** | **0.36%** | **0.38%** | **0.37%** | **0.34%** | **0.59%** | **0.62%** |
| + - - - + | 3 | **0.43%** | **0.38%** | **0.39%** | **0.50%** | **0.42%** | **0.37%** | **0.33%** | **0.29%** |
| - + - - + | 3 | **0.04%** | **0.34%** | **0.38%** | **0.47%** | **0.38%** | **0.31%** | **0.29%** | **0.38%** |
| - + - + - | 3 | **0.11%** | **0.16%** | **0.18%** | **0.16%** | **0.17%** | **0.19%** | **0.32%** | **0.34%** |
| - - - - + | 4 | **3.57%** | **3.30%** | **2.76%** | **2.71%** | **1.93%** | **1.12%** | **0.58%** | **0.37%** |
| - - - + - | 4 | **1.12%** | **1.81%** | **2.15%** | **1.89%** | **1.57%** | **1.36%** | **0.84%** | **0.49%** |
| - - + - - | 4 | **0.93%** | **0.87%** | **0.99%** | **0.66%** | **0.76%** | **0.60%** | **0.63%** | **0.32%** |
| - + - - - | 4 | **0.32%** | **0.41%** | **0.45%** | **0.36%** | **0.42%** | **0.34%** | **0.38%** | **0.55%** |
| + - - - - | 4 | **2.35%** | **2.24%** | **2.33%** | **1.74%** | **1.54%** | **1.20%** | **1.23%** | **1.13%** |
| - - - - - | 5 | **19.79%** | **18.51%** | **16.01%** | **11.94%** | **9.25%** | **6.50%** | **3.82%** | **2.84%** |

The pattern corresponds to the EQ-5D profile, where the first digit represents Mobility, the second Self-care, the third Usual activities, the fourth Pain/Discomfort and the fifth Anxiety/Depression.

**Feasibility properties of the EQ-5D-3L and 5L: Evidence from the GP Patient Survey**

Appendix

Table A2: Distribution of EQ-5D-3L profile missing value patterns by age groups.

| Pattern | Missings | EQ-5D-3L | | | | | | | |
| --- | --- | --- | --- | --- | --- | --- | --- | --- | --- |
|  |  | 18-24 years | 25-34 years | 35-44 years | 45-54 years | 55-64 years | 65-74 years | 75-84 years | 85 or over |
| + + + + - | 1 | **21.88%** | **19.93%** | **22.37%** | **25.64%** | **29.96%** | **33.00%** | **32.11%** | **30.08%** |
| + + + - + | 1 | **11.25%** | **12.36%** | **12.84%** | **13.75%** | **12.97%** | **12.05%** | **11.52%** | **10.10%** |
| + + - + + | 1 | **9.98%** | **10.01%** | **9.12%** | **8.07%** | **7.26%** | **7.14%** | **7.89%** | **9.66%** |
| + - + + + | 1 | **6.74%** | **6.93%** | **7.00%** | **7.74%** | **8.88%** | **8.50%** | **9.33%** | **11.77%** |
| - + + + + | 1 | **10.12%** | **10.06%** | **9.27%** | **9.70%** | **9.62%** | **8.49%** | **7.00%** | **6.44%** |
| + - + + - | 2 | **0.77%** | **0.70%** | **0.87%** | **1.36%** | **2.06%** | **2.65%** | **3.53%** | **3.86%** |
| + + + - - | 2 | **1.54%** | **2.54%** | **2.96%** | **2.98%** | **3.20%** | **3.80%** | **4.40%** | **4.73%** |
| - - + + + | 2 | **1.60%** | **2.15%** | **2.78%** | **2.50%** | **2.32%** | **1.86%** | **1.60%** | **0.92%** |
| + + - + - | 2 | **0.68%** | **0.87%** | **0.85%** | **1.13%** | **1.25%** | **1.58%** | **2.34%** | **3.20%** |
| + - - + + | 2 | **0.80%** | **0.80%** | **0.83%** | **1.04%** | **1.12%** | **1.11%** | **1.58%** | **1.53%** |
| - + + + - | 2 | **0.92%** | **0.83%** | **0.94%** | **1.16%** | **1.04%** | **1.51%** | **1.27%** | **1.17%** |
| + + - - + | 2 | **1.04%** | **0.86%** | **1.07%** | **0.91%** | **0.75%** | **0.65%** | **0.92%** | **1.12%** |
| - + + - + | 2 | **0.95%** | **0.63%** | **0.70%** | **0.76%** | **0.59%** | **0.62%** | **0.72%** | **0.82%** |
| - + - + + | 2 | **0.77%** | **0.61%** | **0.47%** | **0.47%** | **0.55%** | **0.60%** | **0.49%** | **0.57%** |
| + - + - + | 2 | **0.33%** | **0.55%** | **0.79%** | **0.55%** | **0.58%** | **0.56%** | **0.65%** | **1.22%** |
| + - - + - | 3 | **0.36%** | **0.57%** | **0.51%** | **0.75%** | **0.88%** | **1.20%** | **1.58%** | **1.58%** |
| - - - + + | 3 | **0.95%** | **1.15%** | **1.35%** | **1.50%** | **1.09%** | **0.79%** | **0.60%** | **0.45%** |
| - - + + - | 3 | **0.59%** | **0.82%** | **0.74%** | **0.91%** | **0.93%** | **0.89%** | **0.76%** | **0.57%** |
| + + - - - | 3 | **0.95%** | **0.80%** | **1.19%** | **0.91%** | **0.68%** | **0.85%** | **1.34%** | **1.76%** |
| + - + - - | 3 | **0.48%** | **0.28%** | **0.44%** | **0.51%** | **0.42%** | **0.51%** | **0.72%** | **1.05%** |
| - - + - + | 3 | **0.77%** | **0.65%** | **0.96%** | **0.66%** | **0.61%** | **0.41%** | **0.36%** | **0.24%** |
| - + + - - | 3 | **0.36%** | **0.45%** | **0.46%** | **0.34%** | **0.35%** | **0.51%** | **0.47%** | **0.53%** |
| + - - - + | 3 | **0.30%** | **0.25%** | **0.33%** | **0.43%** | **0.41%** | **0.33%** | **0.35%** | **0.38%** |
| - + - - + | 3 | **0.27%** | **0.29%** | **0.18%** | **0.24%** | **0.13%** | **0.11%** | **0.16%** | **0.26%** |
| - + - + - | 3 | **0.09%** | **0.21%** | **0.18%** | **0.20%** | **0.21%** | **0.30%** | **0.31%** | **0.37%** |
| - - - - + | 4 | **2.20%** | **2.77%** | **2.65%** | **2.01%** | **1.22%** | **0.80%** | **0.39%** | **0.34%** |
| - - - + - | 4 | **0.92%** | **1.63%** | **1.53%** | **1.26%** | **1.43%** | **1.11%** | **0.83%** | **0.48%** |
| - - + - - | 4 | **1.54%** | **1.32%** | **1.09%** | **1.04%** | **0.86%** | **0.79%** | **0.67%** | **0.33%** |
| - + - - - | 4 | **0.59%** | **0.91%** | **0.64%** | **0.45%** | **0.38%** | **0.33%** | **0.53%** | **0.37%** |
| + - - - - | 4 | **1.90%** | **2.19%** | **1.63%** | **1.30%** | **1.28%** | **1.20%** | **1.49%** | **1.55%** |
| - - - - - | 5 | **18.35%** | **15.88%** | **13.26%** | **9.72%** | **6.97%** | **5.76%** | **4.11%** | **2.58%** |

The pattern corresponds to the EQ-5D profile, where the first digit represents Mobility, the second Self-care, the third Usual activities, the fourth Pain/Discomfort and the fifth Anxiety/Depression.
